# Unusual Case of Superior Vena Cava Syndrome Caused by Intravascular Thyroid Metastasis

**DOI:** 10.1155/2017/8675239

**Published:** 2017-09-06

**Authors:** Vanesa Varela Pose, María Patricia Fierro Alanis, Anaberta Bermudez Naveira, Oskarina Lourdes Silva Gonzalez, Jorge Fernandez Noya, Urbano Anido Herranz, Rafael Lopez Lopez

**Affiliations:** ^1^Medical Oncology Department, Complejo Hospitalario Universitario de Santiago de Compostela, Santiago de Compostela, Galicia, Spain; ^2^Nuclear Medicine Department, Complejo Hospitalario Universitario de Santiago de Compostela, Santiago de Compostela, Galicia, Spain; ^3^Radiology Department, Complejo Hospitalario Universitario de Santiago de Compostela, Santiago de Compostela, Galicia, Spain; ^4^Angiology and Vascular Surgery Department, Complejo Hospitalario Universitario de Santiago de Compostela, Santiago de Compostela, Galicia, Spain

## Abstract

Superior cava venous obstruction use to show a typical clinical presentation and a CT scan or even an ultrasonography can be sufficient to achieve an accurate diagnosis, but in this case, to obtain the final diagnosis, a multimodal assessment is needed. This case report shows a multidisciplinary approach which helped diagnose a complicated case, where conventional diagnostic methods were not enough.

## 1. Introduction

Superior vena cava syndrome (SVCS) is very unusual and associated with thyroid cancer; the most common tumors related to this syndrome are lung cancer, lymphoma, and mediastinal metastases. Only a few cases have been described in the literature [1]. We found in this case an even more rare form of presentation of the superior vena cava syndrome.

## 2. Case Presentation

An 80-year-old woman presented to medical emergency with two days' history of edema in the left arm.

As relevant medical history, she was diagnosed with a follicular thyroid cancer in 2003. A total thyroidectomy was performed, and she needed to be given two ablative doses of I131 [2]. In 2006, a superior lung lobectomy in the right side was carried out after the disease was detected in a PET study of that localization. Two years later, more metastatic lung disease was noticed, but with a slow growth. In 2009, after clear disease progression, sorafenib (400 mg every 12 hours) as a treatment was started (off-label) [3–6]. She has been taken this treatment for over 34 months until lung progression in 2012. At this time, the treatment with sorafenib was interrupted and a second-line treatment with sunitinib (37.5 mg every 24 hours) was initiated [7] (off-label use). Unfortunately, after two months of the therapy modification, the patient went through two necessary surgeries; the first one was precipitated by an intestinal occlusion by adhesions (she had a history of appendectomy) and the second one by an intestinal perforation. She stopped treatment with sunitinib after those events and started follow-up without evidence of disease progression until now.

At this moment, she was admitted in the medical emergency because of the increasing edema in the left upper limb. She also refers to 15 days' history of palpebral edema. She denied worsening of basal dyspnea, chest pain, or recent episodes of hemoptysis. On examination, she was eupneic, and there was evidence of fullness of the head, with remarkable palpebral edema, a collar of stokes and notable edema of the entire left superior member and left breast. Collateral circulation and telangiectasia in the left pectoral were also noticed.

An eco-Doppler was performed by the vascular surgery service with an inadequate exploration of the subclavian vein because of the edema, but it was evidence of permeable veins of the rest of the left upper limb. A contrast-enhanced CT was recommended which was performed with focus on the cervical, thoracic, and axillary area. There were no signs of compressive masses or intravascular filling defects. One week before, an evaluation CT scan for thyroid cancer was performed, and when reviewed at that moment, it was not showing compressive masses or filling defects either.

Despite no proof of SVCS in imaging test, corticosteroids (dexamethasone 4 mg each 8 hours) and low molecular weight heparin at anticoagulant doses were started by the clinical suspicion. The patient was asymptomatic, so she was discharged with a close follow-up in the oncology consultation.

**Figure 1:**
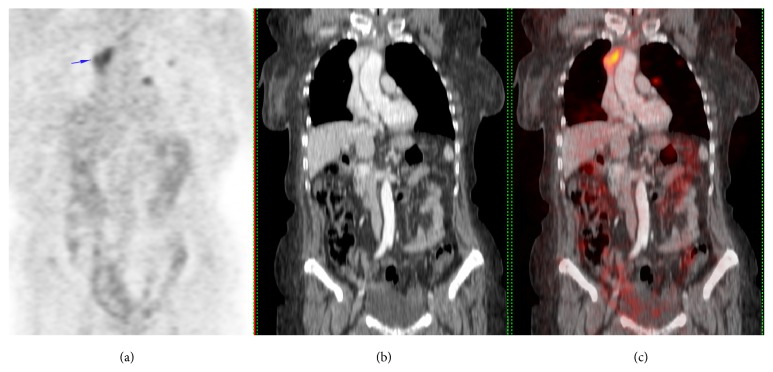
(a) An uptake in the 18F-fluoro-D-glucose PET and (b) small depletion defect in the innominate vein growing towards the superior cava vein, and when we did the fusion with CT scan, (c) intravascular metastasis was found.

After a slight improvement, the patient worsened again and was admitted to the hospital for further examination. On reexamination at this moment, there was notable edema of the head, upper chest, and both upper limbs. However, a clear asymmetry persisted and left upper limb and the left side of the chest were more edematous. During the hospitalization, a PET was performed showing increased activity in the already known lung metastasis and a new image with also increased activity that extended from the thoracic inlet to the anterior mediastinum (Figure 1(a)). The PET was compared with the previous CT (Figures 1(b) and 1(c)) by the radiologist, who reports an intravascular metastasis of 23 × 31 × 45 cm. This metastasis was invading the proximal region of the left brachiocephalic vein with a progressive intravascular growing that filled and expanded the superior vena cava (SVC). Those findings explained the usual clinical signs of the SVCS that were present in our patient, but also the atypical beginning of the case with the asymmetrical edema in the upper limbs.

The case was reviewed by the vascular surgery team which decided on the implantation of two stents to resolve the SVCS. After the extraction of a tissue sample from the SVC invasion, one XL stent was placed in SVC, and another stent was implanted in the left subclavian vein without acute complications.

The patient condition improved within days, and she was released from the hospital. Unfortunately, a few days later, the patient was readmitted to the hospital because of a nosocomial pneumonia which resulted fatally.

## 3. Discussion

The SVCS is the clinical expression of external compression or intrinsic obstruction of the SVC. Malignant conditions remain the leading cause of the SVCS even though in the more recent reviews [8] benign etiologies are increasing with the increasing use of intravascular devices. Among the malignant causes, lung cancer is by far the most frequent cause, followed by lymphomas and metastasis or other tumors, breast cancer, germ cell tumors, sarcomas, and so on [9]. External compression with or without invasion of the SVC represents the most common physiopathology in these cases.

SVCS encompasses various signs and symptoms. The most typical is the swelling of the neck and dyspnea. The edema of the neck is accompanied by a substantial number of cases with facial and upper chest edema and with prominent superficial vessels in this area and jugular venous distention.

The diagnosis of SVC syndrome is made clinically with an accurate clinical history and physical examination, focusing on the duration and the speed of symptoms onset and the history of previous invasive procedures or malignant diseases. After a clinical diagnosis, contrast-enhanced CT is the key examination to find the abnormality underlying the SVCS and its characteristics to plan the best therapeutic strategy in each case [10]. Other imaging techniques that can be used are the contrast-enhanced magnetic resonance or the superior vena cavography and, occasionally, Doppler ultrasound can be helpful.

## 4. Conclusion

The case we present in this paper represents an unusual physiopathology of an SVCS which made the diagnosis confirmation by image a challenge. However, in atypical cases like this, where there are signs not usually associated (like asymmetrical edema) and the CT does not show any justifiable cause for an SVCS, a multimodal assessment can be necessary. In this case, the implications of the vascular surgery team and the Radiology and the Nuclear Medicine Departments allowed not only an accurate diagnosis but also an effective treatment.
